# Challenges and opportunities for risk‐ and systems‐based control of *Listeria monocytogenes* transmission through food

**DOI:** 10.1111/1541-4337.70071

**Published:** 2024-11-28

**Authors:** Alexandra Belias, Samantha Bolten, Martin Wiedmann

**Affiliations:** ^1^ Department of Food Science Cornell University Ithaca New York USA

**Keywords:** environmental monitoring, *Listeria*, ready‐to‐eat, root cause analysis, whole genome sequencing

## Abstract

*Listeria monocytogenes* contamination of ready‐to‐eat (RTE) food products and food‐associated built environments (e.g., processing facilities) represents a food safety issue with major public health and business risk implications. A number of factors make *L. monocytogenes* control a particular challenge, including (i) its frequent presence in different environments, (ii) its propensity for establishing persistence in food‐associated environments, (iii) its ability to grow under a variety of stressful conditions, and (iv) its ability to cause severe illness, particularly in immunocompromised individuals and pregnant people. Key sources of *L. monocytogenes* contamination of RTE foods are food‐associated built environments. However, raw materials can also be an important source, particularly for products without a “kill step” (e.g., fresh produce, raw dairy products, cold‐smoked seafood). While certain RTE foods (e.g., deli meats, soft cheeses, produce) have commonly been linked to listeriosis outbreaks, cases, and recalls, a number of factors will influence the specific public health risk a given RTE food represents, including the likelihood of contamination, ability to support *L. monocytogenes* growth, and consumer‐related factors (including consumption by pregnant or immunocompromised individuals). Consequently, a risk‐based approach presents the most appropriate strategy to minimize the public health and business impact of *L. monocytogenes*. Key challenges to control *L. monocytogenes* include (i) development and implementation of food safety systems that prevent *L. monocytogenes* persistence in food‐associated built environments, (ii) minimizing *L. monocytogenes* contamination of raw material sources, (iii) implementation of effective root cause analysis procedures, (iv) minimizing *L. monocytogenes* growth in finished product, and (v) consumer education.

## 1 INTRODUCTION

*Listeria monocytogenes* is a bacterial foodborne pathogen that causes an estimated 1591 (Scallan et al., 2011) illnesses per year in the United States and an estimated 23,510 (Maertens de Noordhout et al., 2014) illnesses per year worldwide. While the total number of illnesses caused by *L. monocytogenes* is small compared to other foodborne pathogens (e.g., nontyphoidal *Salmonella* spp., which causes an estimated 1,027,561 foodborne illnesses per year in the United States), *L. monocytogenes* has a high case fatality rate of ∼16% (Scallan et al., 2011), making it a serious public health concern. The risk of illness and death caused by *L. monocytogenes* is especially high for pregnant people, elderly individuals, and other individuals with compromised immune systems. *Listeria monocytogenes* can cause an invasive infection with symptoms including sepsis and meningitis in immunocompromised individuals (as well as rarely in healthy individuals) and spontaneous abortions in pregnant people (Schlech, 2019). *Listeria monocytogenes* can also cause gastrointestinal illness in healthy individuals. Additionally, contamination of ready‐to‐eat (RTE) food products with *L. monocytogenes* is a common cause of food recalls, which can damage company reputations and cause substantial financial losses. In fact, there were 90 recalls of foods and beverages in the United States due to *L. monocytogenes* contamination reported between 2022 and 2023 (US Department of Agriculture Food Safety Inspection Service, 2024; US Food and Drug Administration, 2024c). As such, public health officials and the food industry have substantial stakes in reducing contamination of RTE food products with *L. monocytogenes*, as well as in the implementation of other strategies that reduce human listeriosis cases (e.g., reducing the growth of *L. monocytogenes* in foods, education campaigns targeting susceptible consumers).

The need to effectively control *L. monocytogenes* has been further heightened with the broad use of molecular subtyping tools and specifically whole genome sequencing (WGS), which has led to improved detection of listeriosis outbreaks, including small outbreaks (e.g., two to three cases) and/or outbreaks that occur over prolonged time periods (e.g., years) (Moura et al., 2017). More specifically, the US Centers for Disease Control and Prevention (CDC) has been performing WGS of all human *L. monocytogenes* isolates since 2013 (Jackson et al., 2016). Similarly, routine WGS of human clinical *L. monocytogenes* isolates has also been performed by the European Centre for Disease Prevention and Control (ECDC) and the Public Health Agency of Canada (PHAC) since 2014 and 2017, respectively (European Centre for Disease Prevention and Control, 2018; Public Health Agency of Canada, 2019), with other public health agencies around the world also increasingly switching to routine use of WGS for characterization of *L. monocytogenes*. Even with WGS, detailed epidemiological data are still needed to reliably and definitively identify the specific food source responsible for a given outbreak. Importantly, subtyping—specifically WGS data—is also increasingly used by regulatory agencies, including the US Food and Drug Administration (FDA) and the US Department of Agriculture Food Safety and Inspection Service (USDA FSIS), to characterize *Listeria* spp. and *L. monocytogenes* isolates obtained from foods and food‐associated built environments. In some cases, these WGS data may provide evidence for *L. monocytogenes* persistence in food processing facilities, which in some countries and jurisdictions may be used by regulatory agencies to help identify unhygienic conditions in a given facility and can lead to facility shutdowns and recalls, even in the absence of detected finished product contamination.

Key factors that affect the risk of human foodborne listeriosis cases linked to a specific food include (i) initial contamination of the food, (ii) the ability of the food to support *L. monocytogenes* growth, and (iii) the susceptibility of the consumers of a specific food product. As (ii) and (iii) have been detailed in a number of publications and reviews (Buchanan et al., 2017; Farber et al., 2021; Hoelzer et al., 2012; ILSI Research Foundation/Risk Science Institute, Expert Panel on Listeria monocytogenes in Foods, 2005; Pouillot et al., 2015), the review presented here specifically focuses on the risks associated with initial contamination of foods, as well as the challenges the food industry faces in its efforts to reduce the contamination and proliferation of *L. monocytogenes* on foods. Efforts to reduce *L. monocytogenes* contamination of food products are complicated by the fact that this organism (i) is frequently found in a variety of different environments, making introduction into raw materials and processing facilities probable, (ii) is able to survive and grow under adverse environmental conditions, and (iii) has a propensity to establish persistent populations in food‐associated built environments (e.g., processing facilities) and equipment (Ferreira et al., 2014; McClure et al., 1989). Consequently, contamination of food products can occur through a variety of different routes, including natural environments (e.g., for raw materials harvested from nature, such as wild‐caught seafood), primary production environments (e.g., livestock or produce farms), raw materials (e.g., raw milk), and the food‐associated built environments (e.g., processing facilities, retail establishments) and equipment themselves, among others (Ferreira et al., 2014).

The remainder of this article will (i) detail sources of *Listeria*, (ii) discuss public health and business risks associated with *L. monocytogenes* and how to develop and implement risk‐based systems to address these food safety issues, and (iii) outline key control strategies and associated challenges.

## 2 SOURCES OF *Listeria*

**FIGURE 1:**
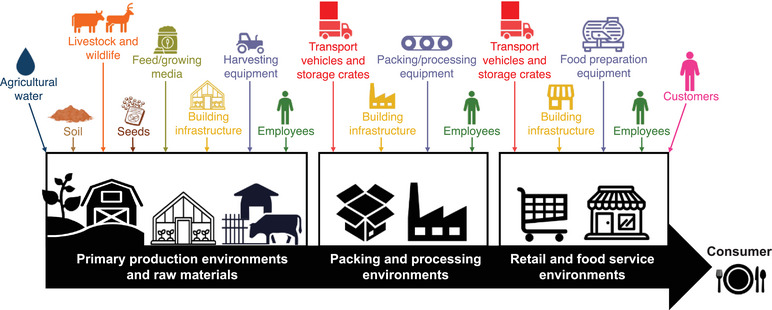
Schematic of potential sources and fomites that can facilitate the introduction of *L. monocytogenes* and other *Listeria* spp. in food‐associated environments, including (i) primary production environments and raw materials, (ii) packing and processing environments, and (ii) retail and food service establishment environments.

While *L. monocytogenes* is the only human pathogen in the genus *Listeria*, testing for the presence of *Listeria* spp. is often used by industry to monitor processing facility environments for the presence of conditions that would facilitate the presence, survival, and/or growth of *L. monocytogenes* (Chapin et al., 2014). *Listeria monocytogenes*, as well as other *Listeria* spp., has been isolated from a wide variety of environments, including from soil, water, feces, and vegetation in the primary production environment (Golden et al., 2019; Nightingale et al., 2004; Strawn, Fortes, et al., 2013; Strawn, Gröhn, et al., 2013; Vilar et al., 2007; Weller et al., 2015a, 2015b); in pristine environments such as national parks (Sauders et al., 2012); in urban environments from sidewalks and automated teller machines (ATMs), among others (Sauders et al., 2012); in processing environments (Ferreira et al., 2014); and in retail and food service environments (Hoelzer et al., 2011). Consequently, many different sources can be responsible for the introduction of *L. monocytogenes* and other *Listeria* spp. into finished product and food‐associated environments, including primary production environments and raw materials, natural environments, and food‐associated built environments such as packinghouses, processing facilities, and retail establishments (Figure 1). Importantly, while employees may act as fomites (El‐Shenawy, 1998; Kerr et al., 1993), there is essentially no evidence that human fecal carriers play a role as sources of *L. monocytogenes* in foods and food‐associated environments (Sauders et al., 2005).

### 2.1 Primary production environments and raw materials

Primary production environments (e.g., farms, fields) and raw materials can play two distinct roles as sources of *Listeria*, including (i) introduction into raw materials that do not undergo a kill step and where *L. monocytogenes* can be carried over into the finished RTE product (e.g., fresh‐cut produce, cold‐smoked seafood, raw milk dairy products) and (ii) introduction into food‐associated environments (e.g., processing facilities) with the potential of subsequent environmental transmission into finished RTE products. Control of *Listeria* in raw materials is hence particularly important for the production of RTE foods that do not involve an effective kill step. Contamination of raw materials can occur from a variety of sources and at a variety of points in the supply chain prior to materials reaching a processing facility (or a retail establishment), including (i) primary production (e.g., produce or livestock farms), including at harvest (e.g., milking equipment, produce harvest equipment), (ii) at an upstream facility (e.g., a storage facility, packinghouse, or slaughterhouse), and (iii) during transportation.

One key supply chain where raw material contamination is of particular concern is whole and fresh‐cut produce. In produce primary production environments, *Listeria* has been isolated from agricultural water sources, soil, vegetation, and wildlife feces (Chapin et al., 2014; Strawn, Fortes, et al., 2013; Strawn, Gröhn, et al., 2013; Weller et al., 2015a, 2015b). With a variety of possible sources, there are a number of transmission pathways that can lead to preharvest produce contamination with *Listeria*. For instance, contaminated irrigation water could directly deposit *Listeria* onto a product or could deposit it into the soil with the possibility of subsequent transmission onto produce (Park et al., 2012; Weller et al., 2015a). Soil could also harbor *Listeria* populations that can be transferred to produce. Finally, wildlife could directly deposit *Listeria*‐contaminated feces onto produce or into the soil. Livestock could present a source of *Listeria* in produce at the preharvest level. The possible role of livestock as a source of *L. monocytogenes* is supported by a listeriosis outbreak in the Maritime Provinces, Canada that was linked to coleslaw likely contaminated from sheep feces (Schlech et al., 1983), as well as the frequent high prevalence of *L. monocytogenes* and other *Listeria* spp. in livestock. In fact, Golden et al. (2019) found a *Listeria* spp. (including *L. monocytogenes*) prevalence of 15.9% (245/1537) and a *L. monocytogenes* prevalence of 1.8% (28/1537) in soil and fecal samples collected from 11 poultry farms in the southeastern United States, further supporting the importance of livestock‐associated sources.

A number of factors can influence how effectively *Listeria* is transferred from soil or other environmental sources to produce. During rain or irrigation events, splashing of soil or wildlife feces can facilitate transfer; in addition, runoff and flooding from adjacent lands can also facilitate contamination of the produce (Pang et al., 2017). Increased wind speed has also been associated with an increased prevalence of *Listeria*, possibly due to increased transfer of *Listeria* from surrounding environments (e.g., farms) (Pang et al., 2017). Unlike *Salmonella* and pathogenic *Escherichia coli*, to date, very few listeriosis outbreaks have been definitively linked to contamination of produce that occurred at the preharvest level. However, given the frequent presence of *L. monocytogenes* in natural and farm environments, it is highly likely that a number of finished product contamination events are linked to the preharvest environment, particularly for products that do not undergo extensive antimicrobial wash treatments or heat treatments. The limited number of outbreaks directly linked to preharvest sources may reflect the fact that many preharvest contamination events (e.g., from wildlife feces or soil splashes) impact small quantities of produce and are thus more likely to lead to individual listeriosis cases rather than outbreaks, although additional information is required to confirm that low levels of *Listeria* are typically present in the preharvest environment. There is, however, growing concern about *Listeria* contamination of produce during harvesting (e.g., via contaminated harvesting equipment), which could lead to more widespread contamination events and thus an increased risk of outbreaks. For example, a recent listeriosis outbreak associated with packaged salads, which resulted in 18 illnesses, 16 hospitalizations, and three deaths across 13 states, was found to be traced back to harvesting equipment contaminated with the outbreak strain (Centers for Disease Control and Prevention, 2022).

Additionally, while less formally characterized compared to traditional preharvest growing environments (e.g., open fields), *Listeria* contamination of produce grown in controlled environment agriculture (CEA) settings can also occur, as evidenced by recent listeriosis outbreaks and *L. monocytogenes* recalls linked to produce such as enoki mushrooms (Centers for Disease Control and Prevention, 2020; US Food and Drug Administration, 2023) and spinach (US Food and Drug Administration, 2024a) grown in CEA settings. In CEA operations, contamination may occur from seeds, water, substrates/other growing media used for CEA production, or the built environment/production equipment, although currently there are limited data surrounding how *Listeria* is spread from these sources to edible or inedible portions of produce (Hamilton et al., 2023). In addition to concerns about *Listeria* contamination, temperature and humidity conditions used to grow produce in CEA settings may also support effective *Listeria* proliferation. In particular, this concern has been raised for enoki mushrooms, where certain growing stages require long periods of exposure to cooler temperatures and high‐humidity conditions that can support *Listeria* growth (Grocholl et al., 2024; Pereira et al., 2023). Importantly, Pereira et al. (2023) noted that enoki mushroom samples collected during a multinational listeriosis outbreak frequently yielded levels of *L. monocytogenes* (i.e., >10^3^ CFU/g) that were higher than those observed in food samples from previous listeriosis outbreaks linked to contamination from packing/processing environments (Chen, Burall, Luo, et al., 2016; Chen, Burall, Macarisin, et al., 2016). These findings may support the likelihood that contamination and proliferation of *L. monocytogenes* on enoki mushrooms occurred during CEA cultivation, as opposed to occurring at later stages of the supply chain. However, given that *L. monocytogenes* has only recently emerged as a food safety concern in CEA systems, more research is needed to fully elucidate the risks of *L. monocytogenes* (and other *Listeria* spp.) contamination and proliferation on produce in CEA primary production environments.

Another supply chain where preharvest contamination is relevant is cold‐smoked seafood. While a number of studies have provided convincing evidence that contamination of finished RTE cold‐smoked products (in particular, cold‐smoked salmon) can be traced back to raw materials (Jahncke et al., 2004), it is rare to identify the specific contamination sources of the raw material (e.g., fish farms, fish slaughter facilities, transport equipment, etc.). Importantly, however, cold‐smoked salmon represents a model for a supply chain that has started to implement innovative approaches to reduce contamination of incoming raw materials, ranging from stringent supplier qualification supported by intensive raw material testing to nonthermal treatments to reduce *Listeria* loads on incoming raw material (Jahncke et al., 2004).

Finally, *L. monocytogenes* contamination of raw materials is also important for raw milk (which is legal for sale in some locations) and other dairy products prepared using raw milk (e.g., raw milk cheese), particularly as *L. monocytogenes* can sometimes be highly prevalent on livestock farms. For instance, Nightingale et al. (2004) found a *L. monocytogenes* prevalence of 20.1% (414/2056) in a survey of 52 ruminant farms where samples were collected of feces, soil, feedstuff, and water. Furthermore, although relatively rare, dairy ruminants can also asymptomatically carry and shed *L. monocytogenes* into their raw milk (Bolten, Ralyea, et al., 2024; Hunt et al., 2012; Papić et al., 2019), in some cases over periods of up to several months (Ricchi et al., 2019). Thus, it is unsurprising that contamination of raw milk and raw milk dairy products continues to represent a major public health concern, with six *L. monocytogenes*‐associated outbreaks and recalls linked to raw milk products being reported in the United States between 2022 and 2023 (Center for Dairy Research, 2024).

In addition to raw materials being a direct source of *Listeria* in finished products and an indirect source (via introduction from farm environments into processing plants), *Listeria* can also establish itself within vehicles and crates used to transport products from primary production environments to packinghouses, processing facilities, or directly to retail. Cross‐contamination during transport is possible whenever the product is exposed to the open environment. To prevent cross‐contamination at this stage, regular cleaning and sanitation of trailers that transport crates and raw materials is thus necessary.

### 2.2 Employees

Employees are sometimes brought up as potential sources or vectors that contribute to the introduction of *Listeria* into the processing environment. However, unlike other pathogens, such as *Salmonella*, it appears unlikely for humans to be fecal carriers of *Listeria* (Sauders et al., 2005). Employees, however, can act as fomites, particularly since *Listeria* can often be found at high frequencies in urban, rural, and natural environments. For example, in a survey of *Listeria* in four urban environments in New York State, Sauders et al. (2012) found the overall *Listeria* spp. (including *L. monocytogenes*) prevalence to be 23.4% (*N* = 907). Once introduced into a facility, *Listeria* can survive over time, particularly if effective food safety and sanitation practices are lacking. In order to prevent the introduction of *Listeria* into the processing environment or onto the product via employees, effective good manufacturing practices (GMPs) must be followed. These GMPs include hand washing and sanitation, wearing gloves, wearing clean coats designated for use only inside of the processing areas, boot washes (or footbaths) at the entrance to the processing areas, and captive footwear policies (e.g., each employee has a set of work boots kept at the facility that may only be used inside the processing areas).

### 2.3 Processing facility and packinghouse environment

The majority of *L. monocytogenes* outbreaks have been linked to RTE foods where contamination originated from processing facilities or packinghouse environments. *Listeria* presence in food processing‐associated environments can conceptually be broken down into two components: (i) introduction into the facility and (ii) the subsequent fate of *Listeria* in a facility, which may include rapid elimination (e.g., through cleaning and sanitation) or survival subsequent to introduction (also often referred to as “persistence”) (Belias et al., 2022). As indicated above, *Listeria* introduction can originate from a number of sources outside a processing facility; preventing introduction through fomites (e.g., people, equipment, etc.) is thus a key part of *Listeria* control programs. Without the proper zoning of equipment and employees, once introduced, *Listeria* can then be moved throughout the facility and can be introduced into a “niche” where it can evade cleaning and sanitation. A lack of sanitary design and proper cleaning and sanitation programs can prevent the elimination of *L. monocytogenes* from the facility (thus facilitating “persistence”). Hence, persistence can typically be traced back to failures associated with prerequisite programs or other nonprocess preventive controls under the Preventive Controls for Human Food Rule such as cleaning and sanitation (US Food and Drug Administration, 2015a). The importance of *L. monocytogenes* persistence in processing facilities has been defined though numerous studies. For instance, Beno et al. (2016) isolated persistent *L. monocytogenes* pulsed‐field gel electrophoresis (PFGE) types (i.e., PFGE types isolated on more than one sampling date) from the processing environments of four out of nine small cheese processing facilities during a survey where environmental samples were collected monthly; 31 of the 57 *L. monocytogenes* strains subjected to PFGE in this study were persistent in a given facility. Furthermore, in a 2011 *L. monocytogenes* outbreak linked to cantaloupes in the United States (McCollum et al., 2013), samples of the brushes used to wash the cantaloupes in the packinghouse yielded *L. monocytogenes* isolates of the same PFGE types as the strains causing illnesses, implicating the brushes as a likely persistent source of contamination in this outbreak. These are two of the many examples that highlight the importance of strong food safety programs at the processing environment level that are designed to control the presence and persistence of *Listeria*; additional examples and more detailed coverage of *Listeria* persistence can be found in a number of reviews on this subject (Belias et al., 2022; Chowdhury & Anand, 2023; Ferreira et al., 2014; Tuytschaever et al., 2023).

Importantly, if suitable conditions exist, *L. monocytogenes* persistence in food packing/processing environments can extend over periods of up to several years. For example, Orsi et al. (2008) found *L. monocytogenes* that persisted in a food processing facility for at least 12 years. Similarly, in a recent listeriosis outbreak associated with RTE dairy products (US Food and Drug Administration, 2024b), *L. monocytogenes* isolates obtained from environmental swabs taken from the implicated dairy processing facility in 2024 matched clinical isolates from the outbreak that were obtained in 2014, indicating that *L. monocytogenes* was likely persistent in this facility for nearly 10 years. Persistent *L. monocytogenes* can be transferred to food contact surfaces and contaminate food products, or a harborage point may develop (i.e., a niche where *Listeria* is present and can continually contaminate product or other areas of the processing environment) within a food contact surface. Some common harborage points include product coolers, forklifts, forklift stops, hollow equipment legs, dead‐end pipes, drains, floor–wall junctures, junctures between equipment legs and the floor, and floor cracks, among other sites that are difficult to clean and sanitize (Simmons & Wiedmann, 2018).

### 2.4 Retail and food services

*Listeria monocytogenes* recalls and outbreaks have also been linked to the contamination originating from retail and food service establishment environments. Similar to processing facilities, the presence of *Listeria* in retail and food service environments is dependent upon (i) its introduction into the environment and (ii) its ability to persist within the environment after it has been introduced. However, compared with a processing environment, retail and food service environments are more open to outside environments, given that there is limited control over what customers bring into the retail space. Therefore, in addition to *Listeria* being introduced on raw materials or with employees, it can also be transported into the retail environment via customers. Hoelzer et al. (2011) conducted a survey of 120 retail delis that were classified as small (<10 employees, *N* = 60) or having failed an inspection (*N* = 60) where they collected samples of food and nonfood contact surfaces, including slicers, utensils, the deli case, floors, drains, and sinks, among others. The *L. monocytogenes* prevalence in these delis ranged from <6% (0/18 positive samples) to 92% (11/12 positive samples); common sites positive for *L. monocytogenes* included milk crates, floors of walk‐in coolers, and drains (Hoelzer et al., 2011). Once introduced into a retail setting, *Listeria* persistence can develop, and cross‐contamination of RTE products can occur (similar to what is observed in processing facilities) if *Listeria* is not eliminated through cleaning and sanitation. As such, frequent and stringent cleaning and sanitation is required to eliminate *Listeria* if introduced into the retail environments; additional deep cleaning and sanitation, which often involves disassembling equipment (e.g., slicers, display cases) as far as possible prior to cleaning and sanitation, is also essential to help effectively eliminate *Listeria* from niches in the equipment (Forauer et al., 2021). In addition, easy‐to‐clean equipment should be used when possible (e.g., using stainless‐steel utensils in place of wooden utensils). Food safety personnel should be included in decision‐making when purchasing or re‐designing equipment to ensure proper hygienic design principles are followed.

## 3 RISK‐BASED APPROACHES TO *L. monocytogenes* CONTROL

*Listeria monocytogenes* contamination of food products and food processing environments can represent both a public health risk and a business (or enterprise) risk, and both need to be managed and minimized. Importantly, currently used approaches to control *L. monocytogenes* can use either risk‐ or hazard‐based approaches; however, risk‐based approaches tend to be more impactful in reducing *L. monocytogenes* illnesses (Barlow et al., 2015).

### 3.1 Public health risks

*Listeria monocytogenes* contamination of RTE foods has been identified as the cause of a number of listeriosis outbreaks worldwide. The risk of a *L. monocytogenes* illness or an outbreak linked to a specific food product is affected by a combination of (i) the likelihood a given food will become contaminated with *L. monocytogenes*, (ii) the food's ability to support the growth of *L. monocytogenes*, (iii) possible inactivation steps before consumption (e.g., cooking), (iv) susceptibility of the products’ consumers, (v) the dose of *L. monocytogenes* ingested from consumption of the contaminated food, and (vi) the virulence potential of the *L. monocytogenes* strain(s) present in the food. The mean *r* (i.e., the probability of a person becoming ill from one cell of *L. monocytogenes*) is estimated to range from 7.9 × 10^−12^ to 9.6 × 10^−9^ depending on the underlying conditions of the person (e.g., age, pregnancy, and other co‐morbidities) (Pouillot et al., 2015). Due to the low probability of illness from a single cell, it is not likely for a product to be contaminated with *L. monocytogenes* at a level high enough to cause illness without *L. monocytogenes* growth in the product. Therefore, the ability of *L. monocytogenes* to grow in a given product plays an important role in its ability to cause an infection. Some RTE foods inherently carry a lower risk of causing *L. monocytogenes* infections given that they either (i) possess intrinsic characteristics (i.e., pH <4.4, water activity <0.92) or (ii) undergo processing, handling, and/or storage conditions that can restrict *L. monocytogenes* growth (US Food and Drug Administration, 2017). However, it is important to note that, while a given food can inherently pose a lower risk due to the inability of *L. monocytogenes* to replicate in it, it is still possible for it to cause illnesses. In particular, the risk of illness increases when the state of the food product is changed in a way that allows it to support the growth of *L. monocytogenes* prior to consumption. This can be illustrated in a multistate listeriosis outbreak associated with caramel apples (Angelo et al., 2017). While both apples and caramel represent foods with limited ability to support *L. monocytogenes* growth, due to the apples’ typical low pH (e.g., <4.0) and caramel's low water activity (<0.80) (Ward et al., 2022), it was hypothesized that the process of piercing the stems of apples with a stick and covering them with caramel could create a microenvironment at the caramel layer–apple surface interface, where access to moisture and nutrients (e.g., from excreted apple juices) would be sufficient to support *L. monocytogenes* growth. This hypothesis has been supported by empirical research studies that observed the growth of *L. monocytogenes* in caramel apples (Glass et al., 2015; Salazar et al., 2016). Therefore, while apples are not an inherently high‐risk product (also due to the containment of nutrients within their waxy skin) as supported by several studies that have not observed growth of *L. monocytogenes* on whole intact apples (Kroft et al., 2022; Salazar et al., 2016; Sheng et al., 2017), downstream processing of the apples in this specific instance increased their risk of causing illness. In addition, the importance of *Listeria* control in frozen vegetables and other frozen products is becoming more apparent. Frozen vegetables are not traditionally considered an RTE product, as consumers are generally instructed to cook these products prior to consumption. However, fruit and vegetable smoothies have become increasingly popular and are often prepared using frozen fruits and vegetables (e.g., berries, spinach, kale), which are generally not cooked prior to blending (Zoellner et al., 2019). While *L. monocytogenes* is unable to grow at freezing temperatures (<0°C), it can survive (Azizoglu et al., 2009). Thus, if these smoothies are not consumed immediately following preparation and are instead left at temperatures that permit *L. monocytogenes* growth (e.g., room temperature, refrigeration temperatures) for sufficient time, *L. monocytogenes* can replicate to a level that increases the likelihood of causing an illness. Similarly, preparation of shakes or smoothies from ice cream with subsequent storage at temperatures that allow *L. monocytogenes* growth can convert a product that would be considered low risk (due to its inability to support *L. monocytogenes* growth at freezing temperatures) into a high‐risk product; this scenario is suspected to have contributed to a 2010–2015 listeriosis outbreak linked to ice cream (Conrad et al., 2023). As such, it is important to consider all potential uses of a product when designing a food safety program, which could include effective cooking labels and instructions as one component of a food safety plan, in order to decrease both the public health and the business risks associated with that product.

In addition, when assessing the public health risk associated with different RTE products, one must consider the target consumers, as some individuals are at a higher risk of infection, with elderly, pregnant, and immunocompromised individuals being particularly susceptible to systemic listeriosis infections. For instance, in a 2010 hospital‐acquired listeriosis outbreak linked to contaminated diced celery, eight of the 10 outbreak case patients were over the age of 65, and all (10/10) case patients were reported to have at least one underlying condition that rendered them immunocompromised or had recently received immunosuppressive treatments that could have increased their susceptibility to listeriosis (Gaul et al., 2013). Moreover, certain products may be consumed at an increased frequency by individuals with an increased risk of acquiring listeriosis (some products may even be specifically targeted toward one of these groups). For example, in the 2010–2015 listeriosis outbreak linked to ice cream, four of the 10 cases were reported to have consumed ice cream products implicated in the outbreak in milkshakes while hospitalized for nonrelated ailments (i.e., they were immunocompromised) (Conrad et al., 2023). As ice cream‐based milkshakes both (i) represent a common calorie‐dense source of nutrition for hospitalized patients, especially those restricted to soft or liquid diets (Okkels et al., 2016) and (ii) may be at higher risk of temperature abuse and *L. monocytogenes* growth compared to whole intact ice cream products, this example illustrates how combinations of different factors can increase the public health risk of a product that may otherwise be considered low risk. Hence, an assessment of the listeriosis‐associated public health risk of an RTE product should also consider how products are handled/manipulated prior to consumption (with a focus on those that increase the potential for *L. monocytogenes* growth) as well as the target consumers of said products and their risk of acquiring foodborne listeriosis. The outcomes of these assessments may indicate the need for additional control strategies, such as specific labeling and detailed preparation instructions.

### 3.2 Business risks

While the public health risk (i.e., the risk of human listeriosis) associated with a product would typically be the driver of *L. monocytogenes*‐focused food safety efforts, individual firms may also want to assess the business and enterprise risks associated with *Listeria*. The predominant business risks associated with *Listeria* typically relate to (i) human disease cases and outbreaks linked to a product and (ii) recalls due to detection of *L. monocytogenes* contamination in product or repeat *L. monocytogenes* (or possibly even repeat *Listeria*) detection in the processing environment. In this context, it is important to note that in some countries (e.g., the United States), any RTE product that tests positive for *L. monocytogenes* would be considered adulterated and hence would have to be recalled, even if the product represents an extremely low public health risk (e.g., sunflower seeds) and even if there are no associated human disease cases. Therefore, *L. monocytogenes* may pose a reasonably high enterprise risk for some products that represent a limited public health risk, which could lead to situations where it may be prudent for firms to make considerable investments into *L. monocytogenes* control, even for products where this organism represents a limited public health risk. Enterprise risks associated with *L. monocytogenes* detection differ considerably based on the regulatory environments. For example, in the United States, there is a so‐called “zero‐tolerance” policy for *L. monocytogenes* in RTE foods. This means no detectable *L. monocytogenes* may be present in two 25‐g samples of FDA‐regulated products and one 25‐g sample of USDA‐regulated products (Archer, 2018). If *L. monocytogenes* is found in any RTE product in the United States, the product must be recalled (if a product is in commerce), regardless of whether any known illnesses have been traced back to the product. If the product is still under the company's control (i.e., not in commerce), the food must be reprocessed with a validated listericidal treatment, repurposed such that it will not be consumed by humans or animals, or destroyed. In addition, it must be determined if other product lots are also potentially contaminated, regardless of whether the products have entered commerce (US Food and Drug Administration, 2017). In the European Union (EU), on the other hand, there are different criteria for RTE foods depending on the potential for *L. monocytogenes* growth. For example, RTE foods that are not able to support the growth of *L. monocytogenes* may have up to 100 CFU/g of *L. monocytogenes* in a given product for the entirety of the product's shelf life (European Commission, 2005). However, for RTE foods that support the growth of *L. monocytogenes*, or RTE foods without data to prove the product's ability to limit *L. monocytogenes* growth to 100 CFU/g at the end of the product's shelf life, *L. monocytogenes* must be absent in five 25‐g samples of the product at the time the product leaves the production facility (European Commission, 2005). Similar to the approach in the EU, in Canada, food products are grouped into two categories (Health Canada, 2023). Category 1 products are those known to support the growth of *L. monocytogenes* and are commonly implicated in outbreaks (e.g., deli‐meats, soft cheeses); for category 1 products, *L. monocytogenes* must be absent in five 25‐g samples (analyzed either separately or composited) of the product. Category 2 products are those that support limited (e.g., fresh‐cut fruits and vegetables) to no growth (e.g., ice cream, hard cheeses) of *L. monocytogenes*; for category 2 products, *L. monocytogenes* levels must be less than 100 CFU/g in five distinct 10‐g samples of the product (Health Canada, 2023).

Quantification of the business risk associated with *L. monocytogenes* should take into account a number of different costs, including (i) costs of illnesses (which a company may be liable for); (ii) costs of product destruction or reprocessing (if permitted), (iii) legal fees; (iv) loss of sales of destroyed product; and (v) loss of future sales due to reputational impacts or (temporary) facility shutdowns. The magnitude of the business risk associated with *L. monocytogenes* can be illustrated by the number of recalls due to *L. monocytogenes* contamination, or suspected contamination. For example, in the United States alone, there were 47 *L. monocytogenes*‐related recalls in 2023, including 18 recalls associated with fresh produce, 12 recalls associated with dairy products, five recalls associated with deli meats or sandwiches/salads containing deli meats, and one recall associated with smoked seafood (US Department of Agriculture Food Safety Inspection Service, 2024; US Food and Drug Administration, 2024c).

## 4 CHALLENGES

Major challenges associated with *L. monocytogenes* control include (i) development and consistent implementation of programs that prevent *L. monocytogenes* introduction and persistence in food‐associated environments (e.g., processing facilities); (ii) management of raw material contamination in products that do not have an effective kill step (e.g., fresh produce, cold‐smoked seafood, raw milk, and raw milk dairy products); (iii) implementation of appropriate root cause analysis (RCA) procedures that allow for identification of sources of environmental and product contamination; and (iv) appropriate use of subtyping methods, including WGS, and appropriate interpretation of the resulting data.

### 4.1 Development and consistent implementation of programs that prevent *L. monocytogenes* introduction and persistence in food‐associated environments

A continued main challenge the industry faces is the development and consistent implementation of programs that (i) prevent *L. monocytogenes* introduction and (ii) prevent *L. monocytogenes* persistence in food‐associated environments (with persistence being defined as survival of a specific *Listeria* subtype in a processing facility over time; see below for details). A related challenge is for regulatory agencies to develop, implement, and enforce regulations that encourage industry to develop and implement stringent programs that minimize the likelihood of *L. monocytogenes* introduction and persistence. For example, regulatory agencies may want to eliminate negative consequences associated with *Listeria* detection in a processing environment to encourage processors to find *Listeria* if it is present. However, some regulations or practices may, unintentionally, provide incentives for companies to not implement stringent environmental and finished product testing strategies. For instance, this may be the case if consequences of positive test results (which are expected due to the frequent presence of *L. monocytogenes* in the environment) are not commensurate to public health risk (e.g., recalls or other severe regulatory consequences due to finding a low level of *L. monocytogenes* in a single finished product sample that does not support its growth or finding *L. monocytogenes* in nonfood contact surfaces in a processing facility). In addition, an allowable level for *L. monocytogenes* in finished products (e.g., those that do not support *Listeria* growth) may incentivize companies to more aggressively test, improving their ability to identify contamination in the processing facility or in raw materials that could lead to finished product contamination with a frequency or at levels likely to cause illness (Farber et al., 2021). More specifically, regulatory agencies could not only set limits of 100 CFU/g for foods that do not support growth (which is consistent with CODEX Alimentarius guidelines [Luber, 2011]) but could also set lower limits using approaches such as three‐class sampling plans, as detailed by Farber et al. (2021).

Minimizing *L. monocytogenes* introduction from outside environments is particularly challenging due to the high prevalence of *L. monocytogenes* in many different environments, as detailed above. Achieving “zero” introduction of *L. monocytogenes* into processing facilities is essentially impossible, particularly if raw materials, which in most cases would have to be expected to at least occasionally be contaminated, are introduced in a facility. Key strategies to minimize the introduction of *L. monocytogenes* include GMPs (e.g., employees should wear clean coats and boots designated for use only within the processing area), regular cleaning and sanitation of trailers used to transport raw materials, regular cleaning and sanitation of forklifts, and regular cleaning and sanitation of creates and bins (including trash bins) that carry materials inside and outside of the facility. However, it is important to note that interventions that introduce additional moisture into the facility (e.g., door foamers and foot baths) can facilitate *L. monocytogenes* growth and survival if not properly maintained (e.g., if appropriate sanitizer concentrations are not consistently maintained). Verifying sanitizer concentrations as well as testing the areas around foot baths and foamers for *Listeria* presence can be useful in identifying a lack of *Listeria* control.

Prevention or management of *L. monocytogenes* persistence in food facilities is a well‐documented issue for the industry. Persistent *Listeria* refers to the *Listeria* that remains in the processing environment for an extended time and is able to survive cleaning and sanitation. Once introduced into the processing environment, *Listeria* can enter niches within the equipment or building infrastructure where cleaners and sanitizers are not able to reach and eliminate its presence, allowing it to become persistent. “Transient *Listeria*,” on the other hand, refers to *Listeria* that is introduced into the processing environment but is subsequently removed during regular cleaning and sanitation activities (Belias et al., 2024). Since *Listeria* is prevalent in a variety of environments, it is expected for *Listeria* to enter the processing environment on occasion. As long as *L. monocytogenes* is quickly (e.g., by the end of a 1‐day shift) removed by cleaning and sanitation and not allowed to survive in a niche within the processing environment, it is unlikely to pose a substantial public health or business risk, thus making transient *L. monocytogenes* a lesser concern compared to persistent *L. monocytogenes*.

An effective *Listeria* sampling program is key to identifying the presence and persistence of *Listeria*. For any RTE foods that are at risk of exposure to the processing facility environment, appropriate testing programs need to include a robust environmental monitoring program and may also include finished product testing, although often at substantially lower frequencies compared to environmental monitoring testing. Finished product testing, particularly if conducted in the absence of a strong environmental monitoring program, is of limited value, as *L. monocytogenes* is often present sporadically and at low levels on food samples, which can make it difficult to identify contaminated products via final product testing. On the other hand, environmental monitoring programs often allow for early detection of potential sources and routes of contamination. A key challenge with environmental monitoring programs, however, is that many lack clear and defined goals, such as validation and verification of *Listeria* control strategies. For instance, routine environmental monitoring programs can be used for verification of *Listeria* control strategies (e.g., cleaning and sanitation). Another important consideration for *Listeria* sampling programs is whether to test for *Listeria* spp. or *L. monocytogenes*. When performing finished product testing, samples should always be tested for *L. monocytogenes*, as a positive result for *Listeria* spp. in a finished product would require further speciation to clearly define the risk associated with the contamination. Meanwhile, when performing environmental monitoring, testing for *Listeria* spp. generally represents the preferred testing strategy, as several nonpathogenic species of *Listeria* often inhabit similar environments as *L. monocytogenes*, and thus, *Listeria* spp. can represent an index organism for *L. monocytogenes* (Chapin et al., 2014). Overall, while strong food safety programs (including environmental monitoring programs) can be costly, they can provide a significant return on investment if they facilitate the identification and elimination of *Listeria* within the processing environment before detection by regulatory agencies or before a public health issue emerges.

In order to identify persistent *Listeria* (and differentiate them from transient *Listeria*), subtyping (e.g., PFGE or WGS) can be used to determine which subtype or strain of *Listeria* is present. If the same or related subtypes are found over time, it is often an indication of persistent *Listeria* or continuous reintroduction of the same subtype. While identifying if a given subtype is persistent or continuously being reintroduced into the environment can be notoriously challenging, certain environmental sampling strategies (e.g., performing preoperational environmental sampling) can help differentiate between these two scenarios. For example, subtype characterization of isolates obtained preoperation (i.e., after cleaning and sanitation, but prior to the next production cycle) can provide strong evidence for persistence (if isolates obtained over time share the same or closely related subtypes) (Bolten, Lott, et al., 2024; Bolten, Ralyea, et al., 2024). On the other hand, identification of isolates that are obtained mid‐operation (e.g., at least 3–4 h into a given production cycle) and share the same or closely related subtypes could also be due to reintroduction.

Importantly, effective food safety programs should be put in place to protect against persistent *Listeria*. These programs must emphasize proper sanitary design of equipment (i.e., elimination of areas within the equipment or facility infrastructure that are difficult to clean and sanitize); a one‐directional flow of employees, equipment, and food products through the processing area; and proper cleaning and sanitation programs, including disassembly of equipment to a level that allows for effective elimination of *Listeria* from niches through cleaning and sanitation activities. In many cases, effective programs may include regular more in‐depth cleaning and sanitation of both equipment (known as “Periodic Equipment Cleaning” [PEC]) and infrastructure (known as “Periodic Infrastructure Cleaning” [PIC]), using validated frequency as well as documentation as part of a Master Sanitation schedule.

In addition to persistent *Listeria* and transient *Listeria*, there is a scenario that can be labeled “persistent transient *Listeria*,” which refers to the continual reintroduction of *Listeria*, representing a single or multiple different subtypes, at a given site or area in the processing environment (Belias et al., 2024). While this scenario may not be as much of a concern as persistent *Listeria*, the continuous reintroduction of *Listeria* to a given area also indicates a lack of proper *Listeria* control. Persistent transient *Listeria* is likely to be introduced into the processing environment with raw materials, crates, and employees, among other routes. In order to reduce the prevalence of persistent transient *Listeria*, more frequent cleaning and sanitation, improved supplier verification, and additional controls to prevent employees from tracking *Listeria* into the processing environment (e.g., captive footwear programs) should be considered.

### 4.2 Management of raw material contamination in products that do not have an effective kill step

While *Listeria* can be easily inactivated by heat, there are a number of products and raw materials that do not receive an effective kill step during processing. Fresh produce, cold‐smoked seafood products, and raw milk dairy products represent examples of commonly consumed RTE products that do not undergo a kill step as part of their processing. For these products in particular, robust supplier verification programs for raw materials are essential to reduce the likelihood that raw materials lead to contamination of the final product. This should include verifying that a supplier has implemented (i) an effective environmental monitoring program if appropriate (e.g., the supplier runs the product through a packinghouse or processing facility) and (ii) thorough cleaning and sanitation programs. In addition, nonthermal and thermal heat treatments that reduce *L. monocytogenes* (although not at the level of a “kill step,” which is typically defined as a 5‐log reduction) can be used. For example, cold‐smoked seafood producers may use antimicrobial washes of incoming raw materials or the addition of antimicrobial treatments (e.g., nisin) to final products to reduce *Listeria* levels and growth (Jahncke et al., 2004). Produce packinghouses and fresh‐cut facilities may also use antimicrobials in wash water to reduce cross‐contamination between produce items (Gil et al., 2009).

### 4.3 Implementation of appropriate RCA procedures that allow for the identification of sources of environmental and product contamination

Since *L. monocytogenes* contamination can originate from a variety of sources and can be facilitated by a number of practices (or lack of practices), identifying the sources of *L. monocytogenes* found in finished products or the environment, as well as contributing factors (e.g., improper execution of preharvest risk assessments, deficiencies in sanitation standard operating procedures [SSOPs]), can be difficult. While a formal well‐defined RCA approach provides one of the most effective ways to define the root cause of a *Listeria* “issue,” implementing good RCA procedures remains a challenge for many companies. RCA is a strategy that aims to identify the true or initial cause of a final event, such that without this initial cause, the final event could not occur. Therefore, using RCA pivots corrective actions from being responsive in nature to being preventive (i.e., with proper RCA, similar problems will be prevented from happening in the future). In particular, RCA can be used to provide a more systematic method for identifying and controlling the presence and persistence of *Listeria* in the food supply chain. For instance, if a persistent *Listeria* subtype is present in a given processing environment, RCA should be performed to (i) identify the source of the persistent *L. monocytogenes*, (ii) eliminate this persistent *L. monocytogenes*, and (iii) identify a control strategy that will prevent similar instances of *Listeria* persistence in the future. In order to perform an RCA, a multidisciplinary team (e.g., someone from quality and food safety, maintenance, and operations) should be formed. Due to the complexity of many food safety problems, getting input from a diverse set of thinkers can help to identify novel causes, as well as innovative corrective actions. Once assembled, the team should clearly define the problem and discuss what information is needed to help solve the problem; the required additional information or data should then be gathered. There are a variety of techniques that can then be used to identify root causes, including fishbone diagrams, the “five whys” technique, change analysis, and fault tree analysis, among others (The PEW Charitable Trust, 2020a); each technique can be used on its own or in combination, and each technique may be most appropriate for different situations. For example, with respect to conducting an RCA related to a *Listeria* contamination event, one might opt to first use fishbone diagrams to visualize all possible components of a given problem, followed by the “five whys” technique (i.e., continually asking why some event or practice occurred, or is the way it is, until reaching the root cause) to further identify the root cause associated with each bone of the diagram that has been deemed important. Some of these RCA techniques have been successfully utilized in a handful of instances (Belias et al., 2021, 2024; US Food and Drug Administration, 2011) toward identifying root causes of *Listeria* contamination in produce packinghouses (Table 1) and may be similarly employed in other food industry sectors to improve management of *Listeria*, as well as other food safety‐related business and enterprise risks.

**TABLE 1:** Key government/industry reports and research studies that utilized root cause analysis (RCA) techniques to aid in controlling *Listeria* contamination and transmission in food.

Source type (Reference)	Purpose of RCA	Personnel represented on the RCA team	RCA technique(s) used	Potential contributing factors for *Listeria* contamination identified by RCA	Outcomes of RCA
Government environmental assessment (US Food and Drug Administration, 2011)	To identify root causes of a *L. monocytogenes* outbreak associated with cantaloupe in 2011	Farm/packinghouse owners; Federal/State inspectors	Fishbone diagram	‐ Lack of sanitary design of various equipment (e.g., roller bar assemblies) used to wash and pack cantaloupes. ‐ Moisture buildup under equipment.	‐ Improved awareness of risks for *Listeria* contamination of fresh produce from the packinghouse environment. ‐ Incorporation of provisions for sanitation of packinghouse infrastructure and equipment in the Produce Safety Rule^a^. ‐ Informed the development of industry guidelines specific to cantaloupe and other netted melons^b^.
Industry report (Blue Bell Creameries, Inc., 2016a, 2016b)	To identify root causes of a *L. monocytogenes* outbreak associated with ice cream between 2010 and 2015	Internal company representatives; outside experts (unspecified)	Undefined^c^	‐ Lack of sanitary design of facility infrastructure and various equipment. ‐ Improper storage of cleaned and sanitized equipment to prevent reintroduction of *Listeria* from other parts of the facility. ‐ Improper employee hygienic practices.	‐ Implementation of interventions focused on enhancing sanitation procedures and hygienic quality of equipment and facility infrastructure. ‐ Improved awareness of risks for *Listeria* contamination of ice cream products from the postpasteurization processing environment. ‐ Increased frequency of regulatory inspections and surveillance of *L. monocytogenes* in US ice cream production facilities^d^.
Research study (Belias et al., 2021)	To identify root causes of reoccurring *Listeria* contamination in the packinghouse environment for one apple packinghouse	Packinghouse manager; quality assurance manager; maintenance manager; two members of the research team	Fishbone diagram; five whys	‐ Frequency of routine cleaning and sanitation activities was not sufficient to control *Listeria*. ‐ Lack of sanitary design of various equipment. ‐ Moisture buildup under equipment.	‐ Implementation of targeted interventions that resulted in reduced *Listeria* contamination at key harborage sites. ‐ A framework outlining how RCA techniques can be applied to identify and control *Listeria* in produce packing/processing facilities.
Research study (Belias et al., 2024)	To identify root causes of persistent *Listeria* in the packinghouse environment for four apple packinghouses	Plant managers; maintenance managers; sanitation managers; food safety and quality managers; two members of the research team	Fishbone diagram; five whys	‐ Frequency of cleaning and sanitation of forklifts was not sufficient to control *Listeria*. ‐ Lack of sanitary design of facility infrastructure and various equipment. ‐ Improper storage and/or removal of waste products from facility.	‐ Implementation of targeted interventions that resulted in reduced *Listeria* prevalence on forklifts, and reduced persistence on various equipment. ‐ A summary of common factors that can contribute to *Listeria* persistence in produce packing environments, and recommendations for industry on practices that can improve *Listeria* control in produce packinghouses.

^a^ Refers to the *Standards for the Growing, Harvesting, Packing and Holding of Produce for Human Consumption* regulations (21 CFR part 112) established under the FDA Food Safety Modernization Act (US Food and Drug Administration, 2015b).

^b^ See Western Growers Association (2013) for more details.

^c^ Procedures used to conduct RCA were not provided in the technical report.

^d^ See US Food and Drug Administration (2022) for more details.

An RCA for identifying the source of contamination or persistent *Listeria* is typically part of a “for‐cause” investigation that also includes efforts to gather sufficient data to facilitate the RCA. This type of “for‐cause” investigation typically requires an intensified sampling of the implicated parts of the processing environment to identify contaminated sites and contamination sources; this type of sampling often involves the collection of hundreds to thousands of samples. While collection of environmental samples is typically key for a *Listeria* RCA, raw material and finished product testing can also be useful and needed. Importantly, sampling as part of RCAs often represents an iterative process where the RCA identifies possible root causes that require sampling for confirmation (or exclusion), often followed by additional sampling to guide further discussions on the root cause and potential corrective actions to eliminate and prevent similar contamination problems in the future.

In addition to identifying actions needed to correct and prevent the occurrence of future *Listeria* contamination issues at the establishment level, lessons learned from RCAs can sometimes guide or inform further industry‐wide improvements. For example, key findings from the FDA's RCA of a growing/packing operation that was implicated in a 2011 listeriosis outbreak linked to cantaloupe (US Food and Drug Administration, 2011) were used to inform both (i) regulatory requirements for sanitation of equipment and infrastructure used for fresh produce packing and (ii) industry guidance for managing food safety risks during cantaloupe production (The PEW Charitable Trust, 2020b; US Food and Drug Administration, 2015b; Western Growers Association, 2013) (Table 1). Similarly, in response to findings from the FDA's investigation of a 2010–2015 listeriosis outbreak linked to ice cream, and internal RCAs performed by the company implicated in this outbreak (Blue Bell Creameries, Inc., 2016a, 2016b; Conrad et al., 2023), the FDA initiated more frequent inspections and heightened surveillance of *L. monocytogenes* in US ice cream production environments (US Food and Drug Administration, 2022).

### 4.4 Appropriate use of subtyping methods, including whole genome sequencing, and appropriate interpretation of the resulting data

In many parts of the world, subtyping (sometimes referred to as “DNA fingerprinting”) methods, particularly WGS, are increasingly used as part of efforts to manage *L. monocytogenes* (Alegbeleye & Sant'Ana, 2020; Jackson et al., 2016). These methods may be used by either (i) an individual company or (ii) regulatory and public health agencies. For example, individual companies may perform subtyping of all *L. monocytogenes* or all *Listeria* spp. isolates that are obtained as part of their routine environmental monitoring programs. Routine subtyping of all isolates helps companies to identify persistent contamination, particularly if positive test results are only sporadically obtained and subtyping is needed to determine whether two positive samples represent contamination with the same *Listeria* or independent events. In addition, some companies do not perform routine subtyping but may perform subtyping only as part of investigations and RCA efforts. Many companies experience challenges with the use of molecular subtyping methods, including (i) the decisions of whether and when to perform subtyping, (ii) the decision of which subtyping method to use, and (iii) performing and interpreting the data outputs (particularly for WGS). The decisions of whether and when to perform subtyping are complex and involve a number of considerations (e.g., regulatory climate, food safety budget, history of *Listeria* issues, etc.), but companies with a strong food safety culture increasingly use these tools. Companies that require advanced information to successfully identify the root cause of a *Listeria* “issue” also typically use subtyping. As for the selection of subtyping methods, commonly used methods include ribotyping, PFGE, and WGS. While the industry often still uses methods such as PFGE and ribotyping, due to typically lower costs, shorter turnaround times, and fewer legal and liability concerns, WGS is being increasingly used by the industry (Jagadeesan et al., 2019).

Public health and regulatory agencies increasingly use WGS to characterize human and food‐associated *L. monocytogenes* isolates as part of either routine inspections or for‐cause investigations. For example, in the United States, WGS is performed on all human *L. monocytogenes* isolates, as well as on any isolates from foods and food processing environments obtained by either the FDA or USDA FSIS. These WGS data are uploaded into the National Center for Biotechnology Information (NCBI) pathogen detection database (https://www.ncbi.nlm.nih.gov/pathogens/) and hence are publicly available, even though the metadata provided do not typically allow for identification of the facility an isolate was obtained from. As part of this process, isolates from foods and food processing environments are also clustered with closely related human isolates and other isolates from foods and food processing facilities. This clustering, and subsequent follow‐up genome comparisons, can be used to identify (i) possible human cases that may be linked to a product or facility (providing a hypothesis for subsequent epidemiological investigations) or (ii) possible instances where a specific strain may persist in an environment. However, these analyses do not identify definitive linkages, and thus WGS data need to be interpreted in conjunction with epidemiological data (Alegbeleye & Sant'Ana, 2020). Industry often struggles with these data analyses, particularly since they often need to be performed and interpreted rapidly to make correct decisions on recalls, recall scopes, and other matters with considerable public health and business impact. While some guidance documents and reviews on interpretation of WGS have been published (Jagadeesan et al., 2019; Pightling et al., 2018), appropriate interpretation of WGS data and associated decision‐making are not trivial and should typically be conducted in consultation with experts to avoid costly errors and misinterpretations.

## 5 CONCLUSION

*Listeria monocytogenes* and other *Listeria* spp. are prevalent in a variety of environments, including natural and urban environments as well as primary production, processing, and retail environments, among others. As such, there are a variety of points along the supply chain where RTE food products can become contaminated with *L. monocytogenes*. While *L. monocytogenes* poses substantial public health risks, it also poses business risks, including when it is found in RTE products that represent a low risk of human disease (e.g., products that do not support *L. monocytogenes* growth [Farber et al., 2021]). As such, risk‐based stringent *Listeria* control programs should be implemented, which include emphasis on GMPs; regular cleaning and sanitation programs; the use of equipment with sanitary designs (i.e., equipment without niches); and appropriate hygienic zoning (e.g., one‐directional flow of employees, equipment, and food products). In addition, food products and their processing environments should be monitored for *Listeria* presence and persistence; these environmental monitoring programs need to be linked to specific goals (e.g., validation and verification of certain food safety programs, such as sanitation programs). Furthermore, robust supplier verification programs and nonthermal antimicrobial treatments are especially important for RTE products produced without a kill step. The large number of potential sources of *Listeria* throughout the food supply chain makes it difficult to identify the true source of contamination when detected in the environment or products. Therefore, the use of formal and well‐executed RCA for “for‐cause” investigations and subtyping tools is thus essential in investigations of *Listeria* positives in order to not only address the specific issue(s) at hand but also create and implement control measures to prevent similar events from occurring in the future.

## AUTHOR CONTRIBUTIONS

**Alexandra Belias**: Conceptualization; investigation; writing—original draft; writing—review and editing; project administration. **Samantha Bolten**: Investigation; writing—review and editing; visualization. **Martin Wiedmann**: Conceptualization; funding acquisition; writing—review and editing; supervision; resources.

## CONFLICT OF INTEREST STATEMENT

Martin Wiedmann serves as a paid consultant for Neogen Corporation on environmental monitoring for *Listeria*. The other authors declare no conflicts of interest.
